# Dynamics of neutralizing antibodies against COVID-19 Omicron subvariants following breakthrough infection in southwest China between December 2022 and April 2024

**DOI:** 10.1038/s41392-025-02319-3

**Published:** 2025-07-30

**Authors:** Yongquan He, Yi Yin, Yi Zhang, Huiping Yang, Zhiling Jiang, Fang Hao, Taiqiang Zhao, Xiaobin Liu, Yusong Liu, Yong Zeng, Xi Li, Xuemei Chen, Kaiju Xu, Chang Tan, Jie Yang, Li Jiang, Bo Gong, Zhenglin Yang

**Affiliations:** 1 Sichuan Provincial Key Laboratory for Human Disease Gene Study, Center for Medical Genetics, Department of Laboratory Medicine, Sichuan Academy of Medical Sciences & Sichuan Provincial People’s Hospital, University of Electronic Science and Technology of China, Chengdu, Sichuan China; 2 Research Unit for Blindness Prevention of Chinese Academy of Medical Sciences (2019RU026), Sichuan Academy of Medical Sciences & Sichuan Provincial People’s Hospital, University of Electronic Science and Technology of China, Chengdu, Sichuan China; 3 Prenatal Diagnosis Center, Chengdu Women’s and Children’s Central Hospital, School of Medicine, University of Electronic Science and Technology of China, Chengdu, Sichuan China; 4 Institute of Microbiological Detection and Analyses, Sichuan Provincial Center for Disease Control and Prevention, Chengdu, Sichuan China; 5 Health Care Department, School of Medicine, Sichuan Academy of Medical Sciences & Sichuan Provincial People’s Hospital, University of Electronic Science and Technology of China, Chengdu, China; 6 Department of Ophthalmology, Sichuan Academy of Medical Sciences & Sichuan Provincial People’s Hospital, University of Electronic Science and Technology of China, Chengdu, China; 7 School of Medicine, University of Electronic Science and Technology of China, Chengdu, China; 8 Infectious Disease Department, Sichuan Academy of Medical Sciences & Sichuan Provincial People’s Hospital, University of Electronic Science and Technology of China, Chengdu, China; 9 Chuan-Yu Joint key Laboratory for Pathological and Laboratory Medicine, Jinfeng Laboratory, Chongqing, China

**Keywords:** Infectious diseases, Medical research

## Abstract

From December 2022 to January 2023, the SARS-CoV-2 Omicron BA.5/BF.7 variant significantly impacted mainland China. While most COVID-19 patients experienced mild symptoms and were treated as outpatients or at home, some cases progressed to severe illness, necessitating hospitalization or even resulting in death. To better understand this outbreak and forecast future waves as SARS-CoV-2 continues to evolve, it is crucial to assess the titer of neutralizing antibodies (Nab) for evaluating the establishment of an immune barrier. In this study, we investigated the dynamic evolution of humoral immunity following the breakthrough infection wave driven by the SARS-CoV-2 Omicron BA.5/BF.7 variant in southwest China. Over a period of more than one year, we analyzed 3128 serum samples collected monthly to delineate the kinetics of Nab responses in a large cohort. Our data revealed a pronounced surge in Nab titers immediately after the December 2022–January 2023 outbreak, particularly among individuals primed with two or three doses of vaccine. As the epidemic progressed, emerging variants such as XBB.1.5, EG.5, and JN.1 elicited distinct immunological responses and demonstrated varying capacities for immune escape. Our findings underscore the rapid antigenic evolution of SARS-CoV-2 and the consequent challenges in sustaining effective population-level immunity, thereby advocating for continual surveillance and adaptive vaccine immunogen updates.

## Introduction

More than four years after the onset of the COVID-19 pandemic, the continuous evolution and global spread of SARS-CoV-2 variants still pose a profound challenge to public health. The virus’s capacity for rapid genetic diversification has yielded successive waves of variants exhibiting increased transmissibility (Alpha, Delta), pronounced immune escape (Omicron BA.1–BA.5), and distinct pathogenic profiles. Each successive variant has demonstrated combinations of enhanced transmissibility, modified virulence and progressively greater capacity for immune evasion. This rapid genetic diversification has underscored the inadequacy of static vaccine designs and the necessity of adaptive immunization strategies. In response, unprecedented global efforts accelerated COVID-19 vaccine research, development, and deployment, with China pioneering mass vaccination campaigns using inactivated virus vaccines such as CoronaVac (Sinovac) and BBIBP-CorV (Sinopharm), based on the original strains. By late 2020, these vaccines became a cornerstone of China’s strategy to mitigate severe disease and death, a commitment that culminated in the administration of billions of doses by December 2022.^1^ These vaccines, derived from the ancestral Wuhan-Hu-1 strain, achieved high coverage across age groups and markedly reduced severe illness and mortality. Yet, as early as mid-2021, real-world studies began to document waning antibody titers and breakthrough infections, signaling that first-generation immunogens would require enhancement or replacement to maintain community-level defenses.

However, the virus’s rapid mutation rate—particularly within the spike protein—gradually eroded the protective efficacy of first-generation vaccines. This was starkly illustrated in December 2022, when China revised its dynamic zero-COVID strategy. The policy shift coincided with a dramatic surge in breakthrough infections, predominantly driven by the immune-evasive Omicron BA.5 and BF.7 subvariants.^2^ During this period, tens of millions of individuals experienced breakthrough infections driven predominantly by the immune-evasive Omicron BA.5 and BF.7 subvariants, provided a dramatic and natural observation in viral dynamics and immune response.^3,4^ The convergence of high baseline seroprevalence and antigenically drifted viral exposures created a unique framework for dissecting how pre-existing humoral immunity adjusts to iterative antigenic challenges. Epidemiological surveillance during this period revealed not only a transient spike in case counts but also provided an unprecedented opportunity to collect high-frequency serological and cellular data, illuminating the interplay between vaccine-induced memory and variant-specific immune evasion mechanisms.

Immunological protection against viral pathogens is inherently complex and influenced by both the characteristics of the pathogen and the host’s immunological history. For instance, viruses such as measles typically induce long-lasting or even lifelong immunity following vaccination or infection,^5^ whereas seasonal human coronaviruses generally elicit transient responses.^6^ This disparity is largely attributable to the intrinsic properties of RNA viruses, which, due to the absence of proofreading mechanisms during replication, accumulate frequent mutations that drive antigenic drift and necessitate continual vaccine reformulation.^7^ Nabs are widely regarded as a key correlation of protection, tend to wane over time, and the emergence of spike protein variants can markedly reduce the binding efficacy and neutralizing capacity of pre-existing antibodies.^8^ Therefore, a detailed longitudinal analysis of Nab titers is critical not only for understanding individual immune trajectories but also for shedding light on population-level immunity dynamics. Such insights are essential for designing adaptive vaccine strategies and assessing the risk of recurrent epidemic waves driven by emerging variants.

In southwest China, this epidemiological context provided the impetus for our study, wherein we launched a longitudinal cohort study in southwest China, systematically collecting 3128 serum samples from December 2022 to April 2024. With balanced gender representation and documented vaccination histories. The frequent sampling allowed us to capture the temporal evolution of the neutralizing antibody response from the onset of the outbreak through successive phases of viral circulation. As the epidemiological landscape shifted, emerging variants such as XBB.1.5, EG.5, and JN.1 subsequently gained prominence, each displaying unique antigenic profiles and differing capacities to evade the established immune background. Through a comprehensive serological analysis, we quantified neutralizing antibody responses using both ELISA-based and pseudovirus neutralization assays, which allowed us to derive geometric mean titers(GMTs) and assess cross-reactivity among the different subvariants. Demographic factors, including age and gender, were also examined to determine their influence on the observed immune responses. This dynamic scenario offered an unparalleled window into the interplay between primary immune responses triggered by breakthrough infections, the waning of antibodies over time, and the boosting effect potentially induced by re-exposure to related subvariants.

Our study ultimately aims to bridge critical knowledge gaps concerning the durability and breadth of post-infection immunity in a densely vaccinated population confronted by continuously evolving SARS-CoV-2 variants. This has significant implications for public health strategies, suggesting that continuous immunological surveillance coupled with periodic vaccine updates may be necessary to maintain an effective barrier against SARS-CoV-2 transmission and preempt future waves of infection.

## Results

### Infection and evolution of SARS-CoV-2

**Fig. 1:**
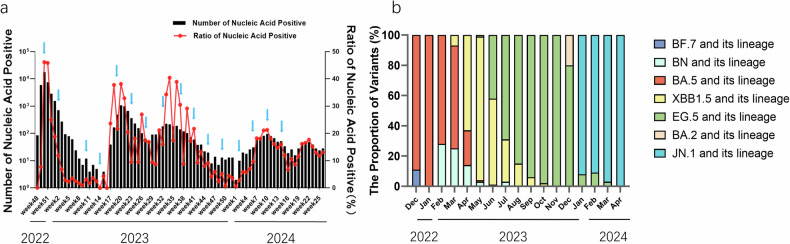
**a** Weekly infected number and ratio of COVID-19 cases in the hospital, starting on December 26, 2022. **b** Proportions of subtypes from the beginning of December 2022. The blue arrow indicates the sampling week

Our outpatient monitoring revealed a rapid increase in infections during the breakthrough infection period, peaking in December 2022 with 17,655 cases reported in week 50. This was followed by two smaller peaks in May and August of 2023, with 1079 cases in week 20 and 227 cases in week 33, respectively. Subsequently, the infection rate showed a consistent decreasing trend (Fig. 1a). To investigate the causes of the initial outbreak and subsequent smaller peaks, we collected serum samples monthly over more than one year, resulting in a total of 3128 samples. These samples were analyzed to detect Nabs against different variants (Table 1). During the breakthrough infection period that started in December 2022, BA.5 was the predominant strain in Chengdu, accounting for 100% of cases in Jan.2023. However, by April 2023, XBB.1.5 and its lineages had become the dominant variants (63%), with the incidence of BA.5 infection dropping to 1% by May 2023. In July 2023, EG.5 and its lineages emerged as the main variants (68%), while JN.1 became the dominant strain at the beginning of 2024 (84%; Fig. 1b).

**Table 1:** Number of detected samples monthly

Year	Month	Detected samples	Vaccination background				Gender	
			NO vaccine	1 Dose	2 Dose	3 Dose	Male	Female
2022	12	272	64	9	57	142	120	152
	1	288	73	6	42	167	139	149
	3	184	33	1	18	132	87	97
	4	192	30	11	29	122	113	79
	5	184	11	3	30	140	95	89
	6	184	9	11	27	137	75	109
2023	7	184	15	4	25	140	76	108
	8	184	28	0	22	134	87	97
	9	184	23	6	18	137	111	73
	10	184	20	1	21	142	82	102
	11	184	9	7	25	143	110	74
	12	184	31	7	31	115	117	67
2024	1	168	58	5	22	83	81	87
	2	184	75	1	20	88	93	91
	3	184	51	5	17	111	95	89
	4	184	25	4	31	124	108	76

### Dynamic humoral responses to different SARS-CoV-2 strains

**Fig. 2:**
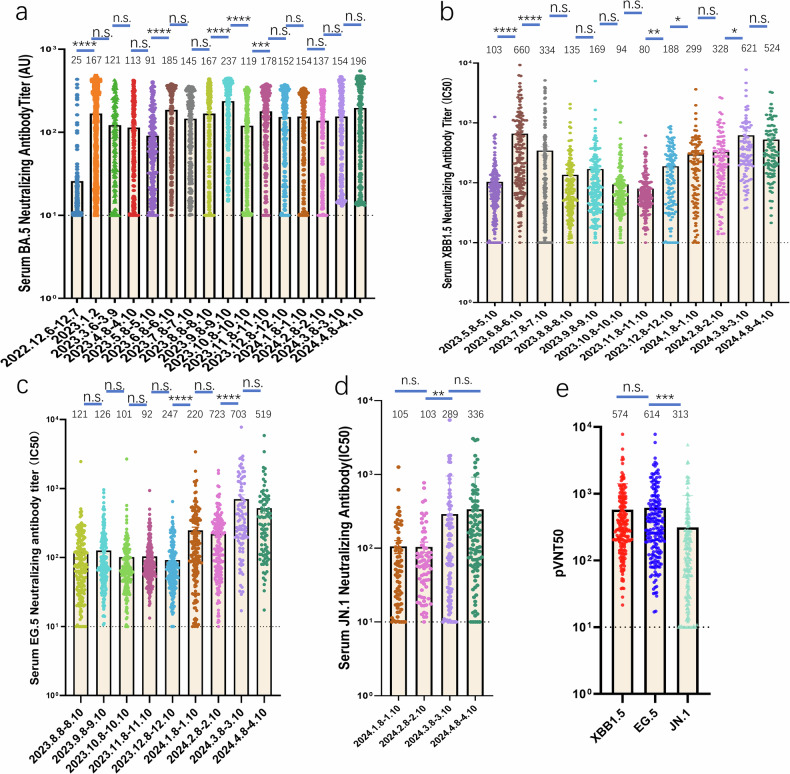
The dynamic changes in neutralizing antibodies against **a** BA.5, **b** XBB.1.5, **c**, and **d** JN.1is also shown. **e** Integrated data on Nabs for XBB.1.5, EG.5 and JN.1 from March and April 2024. The values of the geometric mean titres (GMTs) of each group are indicated in numbers on the top of each panel. BA.5 titers were determined via an ELISA-based assay, whereas neutralizing titers for XBB.1.5, EG.5, and JN.1 were measured using pseudovirus assays. The dashed line: The Lower Limit of Detection. The *P*-values were compared using Kruskal–Wallis test with Dunn’s multiple comparison correction. Data are represented as mean ± SEM. n.s. not significant, *P < 0.05, **P < 0.01, ***P < 0.001, ****P < 0.0001

We detected Nabs against BA.5 in December 2022 (Fig. 2a) via an ELISA-based assay. In December 2022, the geometric mean titer (GMT) of Nabs against BA.5 was as low cases in the negative controls (below 25), suggesting a lack of effective Nabs in the population. This is likely to explain why so many people contracted COVID-19 simultaneously during this period. Research has shown that fully vaccinated individuals could produce more Nabs during breakthrough infections.^9,10^ Accordingly, we analyzed the Nab titers based on vaccination status (Supplementary Fig. S1) from samples collected during breakthrough infections. The results showed that individuals in the 2-Dose and 3-Dose vaccinated groups had higher Nab titers compared to the unvaccinated group in January 2023. This observation indicates that prior vaccination may elicit a more robust anamnestic immune response upon initial exposure to SARS-CoV-2, even in cases of breakthrough infection. However, this enhanced immune activation does not appear to consistently extend to subsequent infections with emerging variants. By January 2023, most people had recovered and exhibited the highest Nab titers, showing a sevenfold increase compared with December 2022. Nabs against XBB.1.5 were tested starting in May 2023 (Fig. 2b) using pseudovirus assays, two months after the strain’s first detection in March 2023. Similarly, Nabs against EG.5 and JN.1 were tested starting in August 2023 (Fig. 2c) and January 2024 (Fig. 2d), respectively. Over the following months, Nab titers gradually declined across the population. The titers against BA.5 and XBB.1.5 rose when infection rates increased, peaking at 40.45% in week 34, with notable increases in June, September, and November 2023 (a 1-2-fold increase compared to May 2023).This pattern suggests that reinfection with different strains induced cross-immunity, as these strains are closely related on the phylogenetic tree.^11^The GMT of Nabs against JN.1 remained relatively stable between January and February 2024 (from 134 to 133), but doubled rapidly in the following months. Simultaneously, the titers of Nabs against XBB.1.5 and EG.5 increased significantly (a 3- to 7-fold increase compared to December 2023). To better understand JN.1’s neutralization evasion abilities, we compared its Nab titers with those of XBB.1.5, EG.5, and JN.1 using data collected in March and April 2024 (Fig. 2e). The GMT for JN.1 was only half that of XBB.1.5 or EG.5. The lower neutralization titer against JN.1 may explain why JN.1 has emerged as a globally dominant variant compared to other variants.

### Correlation between age and humoral responses to different SARS-CoV-2 strains

**Fig. 3:**
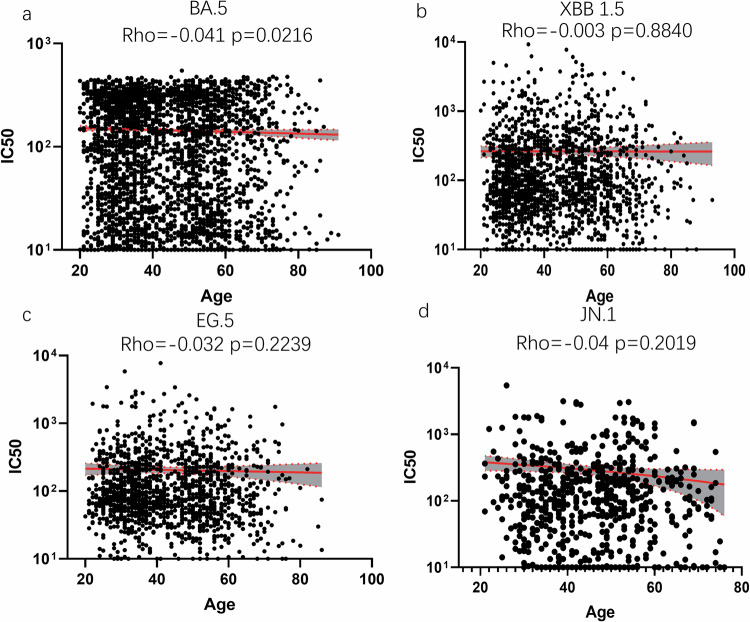
Correlation between age and neutralization antibodies against **a** BA.5, **b** XBB.1.5, **c** and **d** JN.1. Spearman rank correlation coefficients and two-tailed *P*-values were shown at the left upper

We then examined the relationship between age and neutralization titers against Omicron subvariants. The Nab titer showed a negative correlation with age for BA.5 (rho = −0.0412, *p* = 0.02, Fig. 3a), XBB.1 (rho = −0.0035, *p* = 0.88, Fig. 3b), EG.5 (rho = −0.032, *p* = 0.22, Fig. 3c), and JN.1 (rho = −0.0365, *p* = 0.33, Fig. 3d) subvariants. Although all of them showed a negative correlation, statistical analysis revealed that the correlation between age and titers was not significant for most SARS-CoV-2 strains, except for BA.5. These findings suggest that, in our cohort, age is not a robust predictor of Nab titers for the majority of the SARS‑CoV‑2 variants investigated. We also analyzed the relationship between gender and neutralization titers against Omicron subvariants (Supplementary Fig. 2). However, only the BA.5 strain presented gender differences, with females had higher Nab titers against BA.5.

## Discussion

Through large-scale population testing, our study overs valuable insights into the dynamic changes in the Nabs against specific strains of SARS-CoV-2. Monthly monitoring of Nab titers enabled us to thoroughly understand the changes following the BA.5 breakthrough infection period and the subsequent establishment of immunity.^12^ In 2021 and 2022, vaccines maintained sufficient neutralizing antibody levels against the Beta and Delta variants, although additional containment measures, such as behavioral guidelines, also contributed to reducing viral spread. According to a report by the Chinese CDC,^13^ JN.1 became the dominant strain nationwide in the third week of January 2024, accounting for nearly 50% of all cases, and reached 90% by the end of February 2024. During this period, the nationwide COVID-19 positivity rate peaked at 14.3%, slightly lower than the 18% observed in our study. Additionally, we noted a significant decline in the number of patient tests compared to earlier periods (Fig. 1a). These findings suggest that the current pattern of case increases every four to seven months is unlikely to result in peaks exceeding those of previous waves, unless a new strain with significant mutations emerges. JN.1, which emerged in late 2023, is phylogenetically distinct from BA.5.^14^ However, we observed that Nabs against XBB.1.5 and EG.5 increased during the JN.1 wave rather than during the BA.5 wave. This indicates that JN.1 can evade the immune barrier established by prior BA.5 infection.^15,16^ Conversely, vaccines based on XBB.1.5 significantly elevated the titers of Omicron-specific Nabs against JN.1.^17^

Instead of focusing on small cohorts or cross-sectional analyses, we collected hundreds of serum samples each month for over a year to observe the dynamic changes in the titers of Nabs against circulating strains. This approach allowed us to demonstrate antibody responses have evolved in real world scenarios. Although nucleic acid testing and clinical diagnoses indicated no large-scale outbreak after February 2023, infection rates increased periodically every few months. These subsequent cases were predominantly asymptomatic or mildly symptomatic. This underscores the importance of dynamically monitoring viral mutations and changes in Nab levels. RNA viruses, including SARS‑CoV‑2, exhibit high mutation rates due to the limited proofreading capacity of their RNA‑dependent RNA polymerases. In our study, we observed that emerging variants—such as XBB.1.5, EG.5, and JN.1—displayed significantly altered neutralizing antibody titers compared to BA.5. These changes in GMTs closely correlate with specific spike protein mutations that reduce antibody binding and facilitate immune escape.^18,19^ This concern is heightened during periods of immune stress screenings, as evolving strains may render existing antibodies less effective. The continuous evolution of RNA viruses under immune pressure highlights the need for ongoing surveillance. While widespread vaccination and prior SARS‑CoV‑2 infections have undoubtedly raised overall population immunity, the classical concept of herd immunity—which posits that infection spread is contained once a critical immunity threshold is reached—may not fully apply to COVID‑19.^20^ This limitation is due to several factors. First, immunity (whether from vaccination or infection) wanes over time, reducing the duration of protection. Second, the emergence of variants with enhanced immune escape properties further undermines long‑term group protection.^21,22^ For instance, our data demonstrate that even in populations with substantial prior exposure, a decline in cross‑neutralizing antibody titers can coincide with the emergence of new variants, which suggests that the immune barrier is compromised. Highlighting the importance of continuous immunological surveillance and adaptive vaccine strategies. The recurring development of new variants to enhance transmissibility can be seen as a natural process of maximizing their evolutionary fitness. Additionally, the covert spread of SARS-CoV-2 in regions with limited genetic monitoring^23^ and persistent SARS-CoV-2 infections in individuals with weakened immune^24^ systems further facilitate the emergence of new variants. Our findings indicate that monitoring dynamic changes in neutralizing antibody titers can provide early warnings of waning immunity and emerging immune evasion. Although Nab measurements alone are not fully predictive of whether a new variant will become dominant, a significant decline in titers—for example, a 2- to 3-fold reduction compared to levels associated with effective protection—especially when coupled with the emergence of variants that are less efficiently neutralized, suggests a compromised immune barrier. Incorporating such immunological signals with genomic and epidemiological data can inform the timely update of vaccine immunogens. This integrated approach is essential for optimizing vaccination strategies and effectively counteracting evolving viral threats on a global scale.

Numerous studies indicate that advanced age is associated with reduced Nab titers against SARS‑CoV‑2, which may contribute to increased susceptibility to Omicron subvariants among elderly individuals.^25,26^ However, several researchers have also observed that Nab responses exhibit considerable age-related variability. In some cohorts, the decline in antibody titers with age appears minimal—an effect that may be masked by confounding factors such as the vaccine platform used and the time elapsed since vaccination.^27^ Our analysis revealed that, apart from BA.5, there were no statistically significant correlations between age and neutralizing antibody titers for XBB.1.5, EG.5, and JN.1. Even for BA.5, the observed correlation was extremely weak, indicating that age was not a major determinant of Nab titers in our cohort. We also analyzed the impact of gender on Nab generation (Supplementary Fig. 2). The results showed that females had higher Nab titers against BA.5, consistent with previous research suggesting that women develop more robust immune responses to infections and vaccinations and are more prone to autoimmune diseases compared to men.^28,29^ While our study provides a comprehensive analysis of humoral immunity in the general population, it does not specifically include high-risk populations, such as heavy smokers and immunocompromised individuals. These groups are known to exhibit distinct immune responses and may be more vulnerable to emerging SARS-CoV-2 variants.

Overall, our data illustrates the dynamic changes in Nabs following the December 2022 breakthrough infection across a large population over a period of more than a year. Continuous tracking of Nabs against emerging SARS-CoV-2 variants has proven critical for maintaining effective pandemic control strategies, including vaccination. Since the immunogens in earlier vaccines were based on the original strains, they did not provide effective pre-existing immunity and were only moderately effective against Omicron lineages,^30^ As new variants continue to emerge and evolve, the choice of vaccine becomes increasingly vital. Our study demonstrates the critical role of viral evolution in immune escape. Molecular and structural analyses into future research will provide a comprehensive understanding of SARS-CoV-2’s mechanisms of adaptation, guiding vaccine development and therapeutic strategies against emerging variants. Moreover, ongoing monitoring of antibodies within the population is essential for understanding the establishment of immunity and the development of broad-spectrum Nabs.

## Materials and methods

### Ethics approval and consent to participate

This study was performed following the principles of the Helsinki Declaration of the World Medical Association and accepted by the Institutional Review Board and the Ethics Committee of the Sichuan Provincial People’s Hospital (2020-462-2). Participants gave informed consent to participate in the study before taking part.

### Samples collection

Samples were collected beginning with Omicron breakthrough cases in Dec. 2022. in the Chengdu, China. Patients with autoimmune disease, organ failure (liver, kidney, respiratory, etc.), active cancer, long-term bedridden conditions, and similar conditions were excluded. Briefly, Serum samples collected during December 2022 and January 2023 were exclusively obtained from patients recovering from breakthrough infections during the peak of the BA.5 wave. Beginning in March 2023, sample collection criteria expanded to include donors undergoing routine physical examinations rather than exclusively targeting individuals with breakthrough infections. The monthly number of detected samples is listed in Table 1. The serum was isolated after being centrifuged at 3000 rpm for 10 min and stored at −80 °C.

### Virus variants sequencing

The virus was sequenced by the Sichuan Provincial Center for Disease Control and Prevention (CDC), resulting in a total of 742 sequences obtained though the CDC system. All qualified sequences were uploaded to the National Genomics Data Center (https://ngdc.cncb.ac.cn/genbase/, see Dataset Number in the Supplementary Table.1).

### Microplate of anti-SARS-CoV-2 neutralizing antibody titer assay

An ELISA-based kit (ACRO Biosystems) was modified to detect neutralizing antibodies against BA.5. Briefly, serum was mixed with HRP-SARS-CoV-2 Spike RBD and incubated at 37 °C for 1 h. Microtiter plates were coated with the “host cell receptor” angiotensin-converting enzyme 2 (ACE2). Samples containing SARS-CoV-2 neutralizing antibodies blocked the protein–protein reaction between ACE2 and the added (S)-RBD–horseradish peroxidase conjugate. After washing, 100 μL substrate solution was added. The plate was sealed with microplate sealing film and incubated at 37 °C for 20 min. Then, 50 μL of stop solution was added to each well and gently shaken to mix. The absorbance was read at 450 and 630 nm using a microplate reader. Titers are corrected using a standard curve. Standards were used to plot a standard curve by logistic regression.^31^

### Construction and production of variant pseudoviruses

Plasmids encoding the SARS-CoV-2 Omicron sub-lineage spike proteins were constructed. HEK293 T cells were transfected with the indicated spike gene using Polyethylenimine (Polyscience). The cells were cultured overnight at 37 °C in a 5% CO_2_ environment. Subsequently, VSV-G pseudotyped ΔG-luciferase (G*ΔG-luciferase, Kerafast) was used to infect the cells in DMEM at a multiplicity of infection of 5 for 4 h, followed by washing the cells three times with 1 × PBS. The supernatants containing SARS-CoV-2 variant pseudoviruses were harvested 21–64 h after transfection and filtered through a 0.45 μm filter. The supernatants were then aliquoted into 2 mL cryotubes and stored at −80 °C. Luciferase activity was measured to determine the titer of the SARS-CoV-2 variant pseudovirus. 50 μL of supernatants containing SARS-CoV-2 variant pseudoviruses were added to infect 2 × 10^4^ ACE2-293T cells in each well of 96-well plates with 100 μL DMEM and 10% FBS. The cell control well did not receive any pseudovirus. After a 48-hour incubation at 37 °C in a 5% CO_2_ environment, the culture supernatant was gently removed, leaving behind 100 μL in each well. Then, 100 μL of luciferase substrate (REVVITY, britelite plus Reporter Gene Assay System) was added to each well. After a two-minute incubation at room temperature, luminescence was detected using a TECAN Spark multifunctional microplate detector.

### Pseudovirus neutralization assays

Neutralization assays were conducted by incubating pseudoviruses with serial dilutions of sera and measureing the reduction in luciferase gene expression. Briefly, Huh7-ACE2 cells were seeded in a 96-well plate at a concentration of 2 × 10^4^ cells per well. The next day, pseudoviruses were incubated with serial dilutions of the test samples in triplicate for 30 min at 37 °C. The resulting mixture was added to cultured cells and incubated for an additional 48 h. The luminescence was then measured by using the Luciferase Assay System.

### Quantitative and statistical analysis

Statistical analyses were performed using GraphPad Prism 8. Error bars represent the means with standard error. Antibody neutralization values were calculated using a five-parameter dose–response curve in GraphPad Prism, and the geometric mean was calculated for the neutralization results. The Kruskal–Wallis test with Dunn’s multiple comparison correction was performed to compare the neutralization IC50 values. No statistical methods were applied to verify whether the data met the assumptions of the statistical approach.

## Supplementary information

Supplementary Materials-Dataset number

Supplementary Materials

## Data Availability

The data supporting the findings of this study are available from the corresponding author upon reasonable request. The sequences are publicly accessible in the National Genomics Data Center under the accession numbers listed in Supplementary Table 1.

## References

[1] Yang, M. et al. COVID-19 vaccination and concerns regarding vaccine hesitancy after the termination of the zero-COVID policy in China: a nationwide cross-sectional study. *Hum. Vaccines Immunother.* **20**, 2388938 (2024). 10.1080/21645515.2024.2388938 PMC11326449 39140437

[2] Liu, J., Liu, M. & Liang, W. The dynamic COVID-zero strategy in China. *China CDC Wkly.* **4**, 74–75 (2022). 35186372 10.46234/ccdcw2022.015 PMC8837441

[3] Goldberg, E. E., Lin, Q., Romero-Severson, E. O. & Ke, R. Swift and extensive Omicron outbreak in China after sudden exit from ‘zero-COVID' policy. *Nat. Commun.* **14**, 3888 (2023). 37393346 10.1038/s41467-023-39638-4 PMC10314942

[4] He, Y. et al. Clinical characteristics of mild patients with breakthrough infection of Omicron variant in China after relaxing the dynamic zero COVID-19 policy. *Vaccines*. **11**, 968 (2023). 10.3390/vaccines11050968 PMC10224174 37243072

[5] Amanna, I. J., Carlson, N. E. & Slifka, M. K. Duration of humoral immunity to common viral and vaccine antigens. *N. Engl. J. Med.* **357**, 1903–1915 (2007). 17989383 10.1056/NEJMoa066092

[6] Edridge, A. et al. Seasonal coronavirus protective immunity is short-lasting. *Nat. Med.* **26**, 1691–1693 (2020). 32929268 10.1038/s41591-020-1083-1

[7] Duffy, S., Shackelton, L. A. & Holmes, E. C. Rates of evolutionary change in viruses: patterns and determinants. *Nat. Rev. Genet.* **9**, 267–276 (2008). 18319742 10.1038/nrg2323

[8] Alenquer, M. et al. Signatures in SARS-CoV-2 spike protein conferring escape to neutralizing antibodies. *Plos Pathog.* **17**, e1009772 (2021). 34352039 10.1371/journal.ppat.1009772 PMC8341613

[9] Wang, X. et al. Neutralization of SARS-CoV-2 BQ.1.1, CH.1.1, and XBB.1.5 by breakthrough infection sera from previous and recent waves in China. *Cell Discov.* **9**, 64 (2023). 37369648 10.1038/s41421-023-00569-5 PMC10300020

[10] Li, Y. et al. Antibody response assessment of immediate breakthrough infections after zero-COVID policy adjustment in China. *Lancet Reg. Health W. Pac.* **40**, 100945 (2023). 10.1016/j.lanwpc.2023.100945 PMC10684796 38033432

[11] Nham, E. et al. Low neutralizing activities to the Omicron subvariants BN.1 and XBB.1.5 of Sera from the individuals vaccinated with a BA.4/5-containing bivalent mRNA vaccine. *Immune Netw.* **23**, e43 (2023). 38188597 10.4110/in.2023.23.e43 PMC10767551

[12] Anderson, R. M. & May, R. M. Vaccination and herd immunity to infectious diseases. *Nature* **318**, 323–329 (1985). 3906406 10.1038/318323a0

[13] China CDC, Novel Coronavirus Infection. https://www.chinacdc.cn/jkzt/crb/zl/szkb_11803/ (2024).

[14] Kaku, Y. et al. Virological characteristics of the SARS-CoV-2 JN.1 variant. *Lancet Infect. Dis.* **24**, e82 (2024). 38184005 10.1016/S1473-3099(23)00813-7

[15] Yang, S. et al. Fast evolution of SARS-CoV-2 BA.2.86 to JN.1 under heavy immune pressure. *Lancet Infect. Dis.* **24**, e70–e72 (2024). 38109919 10.1016/S1473-3099(23)00744-2

[16] Favresse, J. et al. Neutralizing antibody response to XBB.1.5, BA.2.86, FL.1.5.1, and JN.1 six months after the BNT162b2 bivalent booster. *Int. J. Infect. Dis.* **143**, 107028 (2024). 38583825 10.1016/j.ijid.2024.107028

[17] Abul, Y. et al. Broad immunogenicity to prior SARS-CoV-2 strains and JN.1 variant elicited by XBB.1.5 vaccination in nursing home residents. *GeroScience*, **47**, 1887–1896 (2025). 10.1007/s11357-024-01346-2 PMC11978566 39395130

[18] Li, D. et al. Function and structure of broadly neutralizing antibodies against SARS-CoV-2 Omicron variants isolated from prototype strain infected convalescents. *J. Transl. Med.* **23**, 212 (2025). 39985112 10.1186/s12967-025-06162-6 PMC11844185

[19] Stein, S. C. et al. A human monoclonal antibody neutralizing SARS-CoV-2 Omicron variants containing the L452R mutation. *J. Virol.* **98**, e122324 (2024). 10.1128/jvi.01223-24 PMC11650997 39494911

[20] Anderson, R. M., Heesterbeek, H., Klinkenberg, D. & Hollingsworth, T. D. How will country-based mitigation measures influence the course of the COVID-19 epidemic?. *Lancet* **395**, 931–934 (2020). 32164834 10.1016/S0140-6736(20)30567-5 PMC7158572

[21] Kissler, S. M., Tedijanto, C., Goldstein, E., Grad, Y. H. & Lipsitch, M. Projecting the transmission dynamics of SARS-CoV-2 through the postpandemic period. *Science* **368**, 860–868 (2020). 32291278 10.1126/science.abb5793 PMC7164482

[22] Gostic, K. M. et al. Practical considerations for measuring the effective reproductive number, Rt. *Plos Comput. Biol.* **16**, e1008409 (2020). 33301457 10.1371/journal.pcbi.1008409 PMC7728287

[23] Ghafari, M., Watson, O. J., Karlinsky, A., Ferretti, L. & Katzourakis, A. A framework for reconstructing SARS-CoV-2 transmission dynamics using excess mortality data. *Nat. Commun.* **13**, 3015 (2022). 35641529 10.1038/s41467-022-30711-y PMC9156676

[24] Clark, S. A. et al. SARS-CoV-2 evolution in an immunocompromised host reveals shared neutralization escape mechanisms. *Cell* **184**, 2605–2617 (2021). 33831372 10.1016/j.cell.2021.03.027 PMC7962548

[25] Bates, T. A. et al. Age-dependent neutralization of SARS-CoV-2 and P.1 variant by vaccine immune serum samples. *JAMA* **326**, 868–869 (2021). 34287620 10.1001/jama.2021.11656 PMC8295896

[26] Wang, H. et al. Neutralization against Omicron subvariants after BA.5/BF.7 breakthrough infection weakened as virus evolution and aging despite repeated prototype-based vaccination(1). *Emerg. Microbes Infect.* **12**, 2249121 (2023). 37668156 10.1080/22221751.2023.2249121 PMC10524800

[27] Lee, B. et al. Age-related differences in neutralizing antibody responses against SARS-CoV-2 Delta and Omicron variants in 151 SARS-CoV-2-Naive metropolitan residents boosted with BNT162b2. *J. Appl. Lab. Med.* **9**, 741–751 (2024). 38531067 10.1093/jalm/jfae014

[28] Bunders, M. J. & Altfeld, M. Implications of sex differences in immunity for SARS-CoV-2 pathogenesis and design of therapeutic interventions. *Immunity* **53**, 487–495 (2020). 32853545 10.1016/j.immuni.2020.08.003 PMC7430299

[29] Grzelak, L. et al. Sex differences in the evolution of neutralizing antibodies to severe acute respiratory syndrome Coronavirus 2. *J. Infect. Dis.* **224**, 983–988 (2021). 33693749 10.1093/infdis/jiab127 PMC7989436

[30] Yuan, R. et al. Enhanced immunity against SARS-CoV-2 in returning Chinese individuals. *Hum. Vaccines Immunother.* **20**, 2300208 (2024). 10.1080/21645515.2023.2300208 PMC10793704 38191194

[31] Zedan, H. T. et al. Evaluation of commercially available fully automated and ELISA-based assays for detecting anti-SARS-CoV-2 neutralizing antibodies. *Sci. Rep.* **12**, 19020 (2022). 36347859 10.1038/s41598-022-21317-x PMC9643483

