# Supplementary material for: Dynamics of neutralizing antibodies against COVID-19 Omicron subvariants following breakthrough infection in southwest China between December 2022 and April 2024
Source: Signal Transduct Target Ther. 2025 Jul 30;10:242. doi: 10.1038/s41392-025-02319-3 (PMC12310930; doi:10.1038/s41392-025-02319-3)
Supplement: Supplementary file 2 — Supplementary Materials [file 41392_2025_2319_MOESM2_ESM.docx]

Supplementary Materials for

Dynamics of Neutralizing Antibodies Against COVID-19 Omicron Subvariants Following Breakthrough Infection in Southwest China between December 2022 and April 2024

**Authors:** Yongquan He^1,2*^, Yi Yin ^3*^, Yi Zhang^1,2*^, Huiping Yang ^4*^, Zhiling Jiang^1,2^, Fang Hao ^1,2^, Taiqiang Zhao ^1^, Xiaobin Liu ^5^, Yusong Liu^1^, Yong Zeng^6^, Xi Li^7^, Xuemei Chen^8^, Kaiju Xu ^8^, Chang Tan ^1,2^, Jie Yang^1^, Li Jiang ^1^, Bo Gong^1,2^, Zhenglin Yang^1,2#^

* Yongquan He, Yi Yin, Yi Zhang and Huiping Yang contributed equally to this work

Correspondence to: yangzhenglin@cashq.ac.cn

**This PDF file includes:**

Figures. S1 to S2

Fig.S1


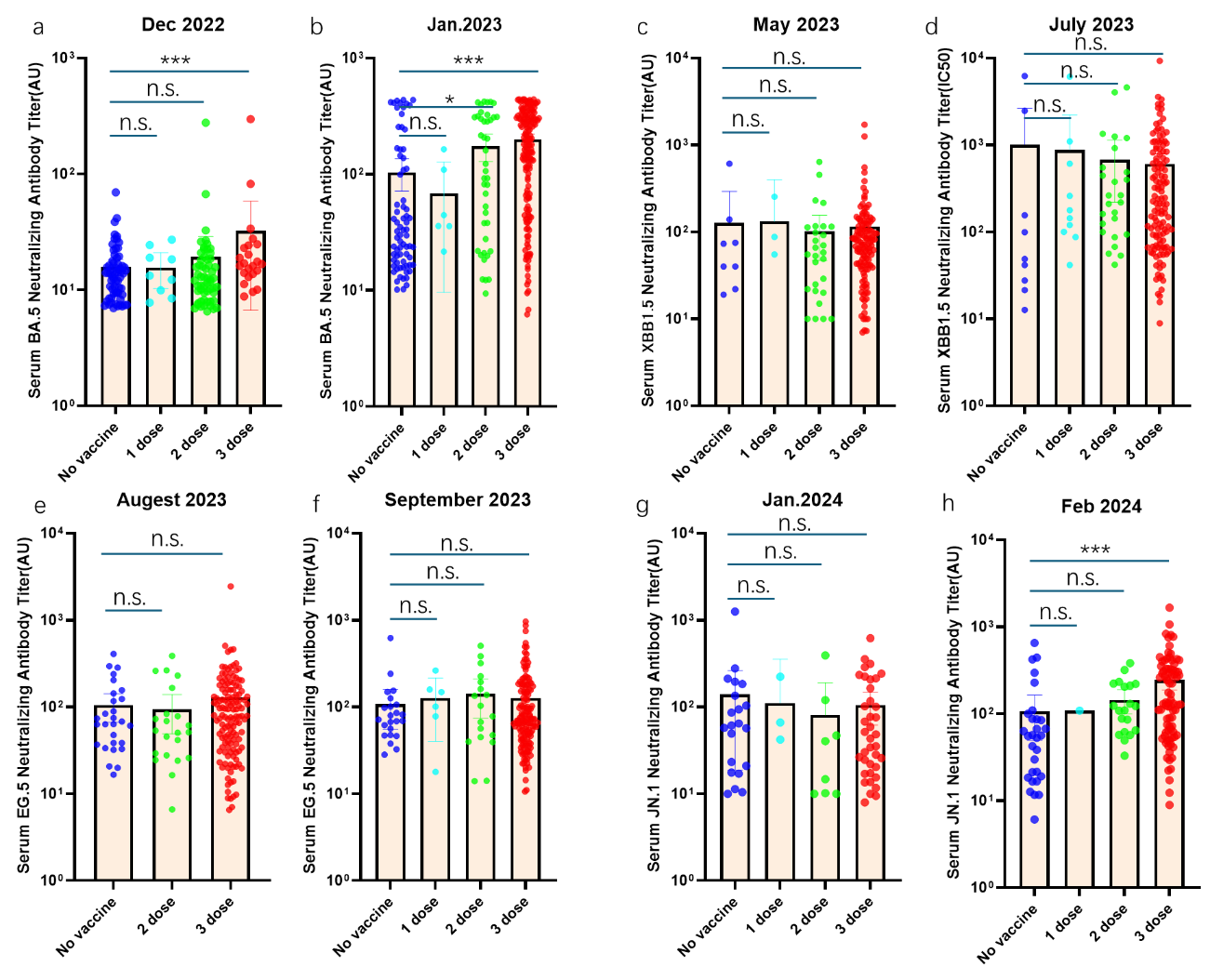


Neutralizing antibody (Nab) titers against SARS-CoV-2 subvariants BA.5(a, b), XBB.1.5(c, d), EG.5(e, f), and JN.1(g,h) across vaccination backgrounds and various time points. The data is presented as mean ± 95% CI. The *P*-values were compared using Kruskal–Wallis test with Dunn’s multiple comparison correction. n.s.: not significant, *P < 0.05, **P < 0.01, ***P < 0.001.

Fig.S2


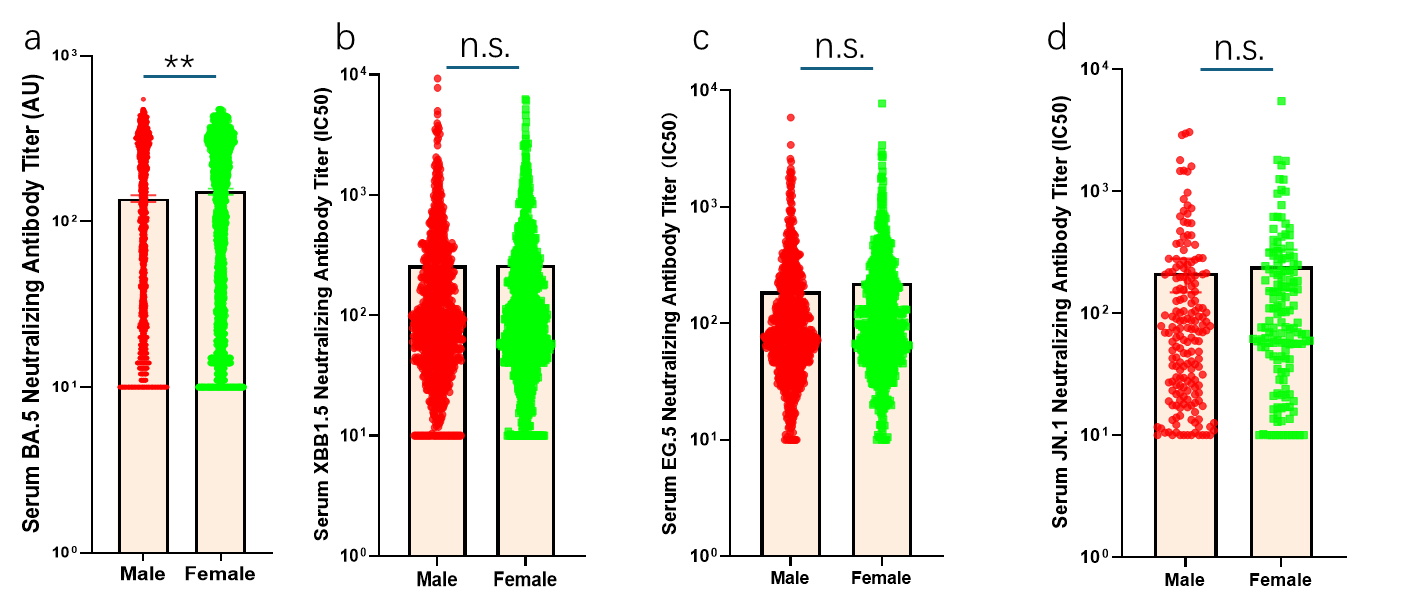


Comparison of the Nabs against (a) BA.5, (b) XBB.1.5, (c), and (d) JN.1 between male and female. Data are mean ± 95% CI. n.s.: not significant, **P < 0.01.
